# Supplementary material for: Uncovering interpretable potential confounders in electronic medical records
Source: Nat Commun. 2022 Feb 23;13:1014. doi: 10.1038/s41467-022-28546-8 (PMC8866497; doi:10.1038/s41467-022-28546-8)
Supplement: Supplementary file 1 — Supplementary Information [file 41467_2022_28546_MOESM1_ESM.pdf]

Supplementary Information

1 Supplementary: Glossary of Structured Covariates

Supplementary Table 1 shows a glossary of the structured features extracted from the EMRs.

Supplementary Table 1: Glossary of structured features extracted from EMR data. The types of variables include: binary (B), categorical(C), and continuous (CT).

Feature		Type	Description
Demographic	race_white	B	1 if patient identifies as race white
	race_black	B	1 if patient identifies as race black
	race_api	B	1 if patient identifies as race asian or pacific islander
	hispanic	B	1 if patient identifies as hispanic
	nonhispanic	B	1 if patient identifies as nonhispanic
	patient_age	C	age split into 7 categories
Cancer Description	clinical_stage	C	clinical stage categories
	tumor_grade	C	tumor grade categories
	grade_unknown	B	1 if patient grade is unknown
	diagnosis_year	CT	year initial diagnosis is recorded

2 Supplementary: Dictionary of Synonymns

We used a dictionary of synonyms based on a list of the most common words to reduce noise in our dataset. Please see the list of synonyms below:

- {"abnormality" → "abnormal", "admission" → "admit", "assistance" → "assistant", "bilateral" → "bilaterally", "bleeding" → "bleed", "consult" → "consultation", "diagnostic" → "diagnosis", "evaluate" → "evaluation", "hx" → "history", "functional"

→ "function", "fu" → "followup", "gentleman" → "man", "disease" → "illness", "imaging" → "image", "improvement" → "improve", "invasion" → "invasive", "action" → "movement", "neurologic" → "neurological", "operative" → "operation", "polyps" → "polyp", "postop" → "postoperative", "pulse" → "rate", "reaction" → "reactive", "refer" → "referral", "removal" → "remove", "resp" → "respiratory", "smoke" → "smoking", "assistance" → "service", "spinal" → "spine", "surgical" → "surgery", "assessment" → "test", "testing" → "test", "therapeutic" → "therapy", "treat" → "treatment", "visualize" → "visual"}

3 Supplementary: Confounders

Confounding is a major challenge when estimating causal effect from observational studies. The structure of confounding can be represented by causal diagrams. In Supplementary Figure 1, we present a series of Directed Acyclic Graphs (DAGs) that show different causal structures with potential confounding, based on the examples in Hernán and Robins [31], Chapter 7. In the following diagrams, we define Y as the outcome, W as the treatment, and X as the covariate that has been identified as a potential confounder.

Supplementary Figure 1a shows the most natural case of confounding. Treatment W is a cause of outcome Y and confounder X is a cause of both W and Y. Therefore, the association between W and Y includes both the direct causal effect and an indirect "backdoor" path from W to Y through X. Conditioning on the confounder X blocks this second path, allowing the accurate estimation of the causal effect of W on Y. Examples of this type of confounder include fixed patient characteristics such as a patient's age or cancer clinical stage. For example, older patients can have worse survival outcomes, so doctors might assign different treatments to older patients; cancer patients with higher clinical stage can also have worse survival outcomes, affecting doctors' treatment decisions.

Our method is designed to uncover and adjust for this type of confounder, as is appropriate.

Supplementary Figure 1b and 1c show different structures where W is a cause of Y, where X is a cause of one and associated with the other through an unmeasured cause U. In these cases, conditioning on confounder X blocks the "backdoor" path between W and Y; in such cases, conditioning on X is necessary to avoid bias in estimating the causal effect of W on Y, since part of the correlation between X and Y arises due to the relationship between (U) and both W and Y. Confounding of type 1b can arise when U represents a type of lung cancer mutation. Even if a mutation test is not performed (and so the mutation is unobserved), the mutation (U) affects the symptoms recorded in the notes (X), as well as the patient outcomes (Y). An example of 1b uncovered from our NSCLC study is text:left.low, tumor location on the left lower lobe. Location of cancer (X) directly effects treatment decisions, and EGFR mutated lung cancer (U) is less likely to be positioned in the lower lobe [38]. For these examples, conditioning on the text describing the cancer location is appropriate, and is necessary if the backdoor path is not blocked by other covariates X.

Supplementary Figure 1d shows a structure where W is a cause of Y, and X is a cause of neither of them. In 1d, conditioning on X will not eliminate the backdoor path between W and Y, but it might reduce its effect. Confounding of type 1d may arise when U represents patient "performance status" (a measure of how the disease impacts the patient's daily living abilities), which is not typically recorded in the patient's chart as a structured field, but can directly effect both treatment decision and survival outcome [41]. Some examples of 1d uncovered from our NSCLC study include text:discomfort, text:alert, and text:attention.

Supplementary Figure 1e shows a structure where W is a cause of X, but not a cause of Y. In such cases, conditioning on X introduces a backdoor path between W and Y.

Confounding of the type shown in 1e can arise when X represents short-term effects post treatment and U represents patient health status. Our study design avoids such cases by only considering pre-treatment covariates.

Supplementary Figure 1f shows a structure where W and Y are causes of X. In this case, the covariate X is referred to as a collider, and conditioning on X introduces a backdoor path between W and Y. Our study design also avoids such cases by only considering pre-treatment covariates.

Supplementary Figure 1g show another structure where W is a cause of Y, and X is a cause of neither of them. Unlike 1d, conditioning on X in 1g opens a backdoor path between W and Y, introducing bias into the causal estimate of the effect of W on Y. Situations such as 1g are a potential limitation of our methodology, but in some cases they can be recognized through inspection and reasoning. Examples of 1g are words selected in earlier iterations of our study such as text:menlo, referring to the location Menlo Park, CA. A patient's education level (U_1) can effect both their treatment preference and also their living location; a patient's socioeconomic status (U_2) can effect their survival outcome and living location. Although text:menlo is associated with treatment and outcome through U_1 and U_2, treating it as a confounder would introduce bias. We were able to filter out some of these terms by selecting only biomedical-related terms. Clinical expertise is needed to avoid scenario 1g.

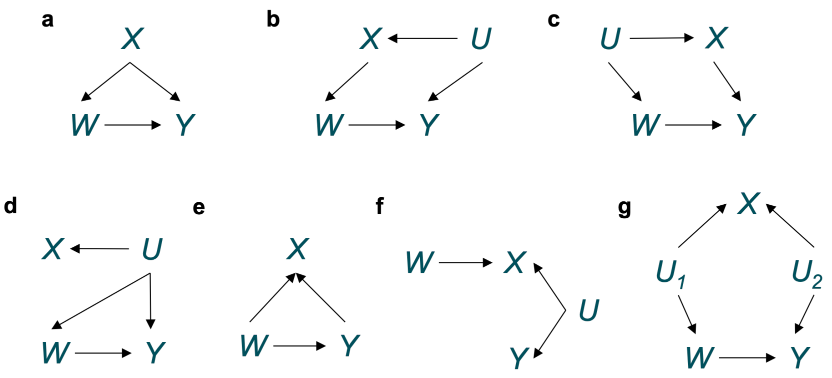

Supplementary Figure 1: Causal diagrams showing different potential cases of confounders. For the diagrams, X represents the features; Y represents the outcome; W represents the treatment; and U, U_1, U_2 are potential unknown confounders.

4 Supplementary: Propensity Score and Covariate Balance Plots

We show the propensity scores and covariate balance plots for each of the results plotted in Figure 3 of the main paper. In Supplementary Figure 2, we show the plots for *structured*. In Supplementary Figure 3, we show the plots for *intersect*. In Supplementary Figure 4, we show the plots for *struct+intersect*.

5 Supplementary: Covariate Correlation

We show the R^2 values for all combinations of the selected covariates for each of the treatment groups. The R^2 is the square of the correlation from linear regression. It measures the proportion of variation in the dependent variable that can be attributed to the independent variable. In Supplementary Table 2, we show the R^2 values for surgery vs. radiation for prostate cancer. In Supplementary Table 3, we show the R^2 values for surgery vs. monitoring for prostate cancer. In Supplementary Table 4, we show the R^2 values for

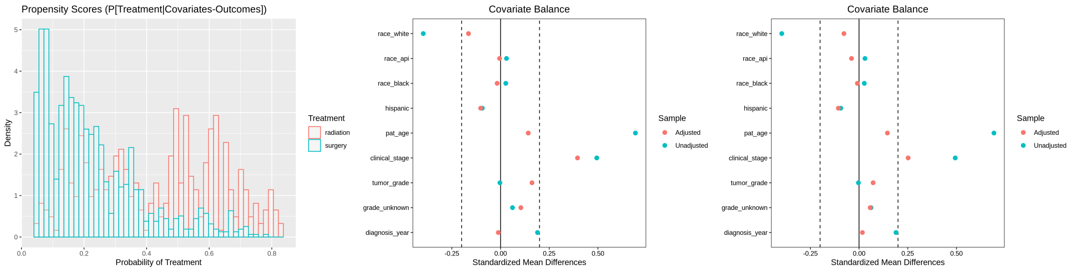

(a) Surgery vs Radiation (prostate, grf)

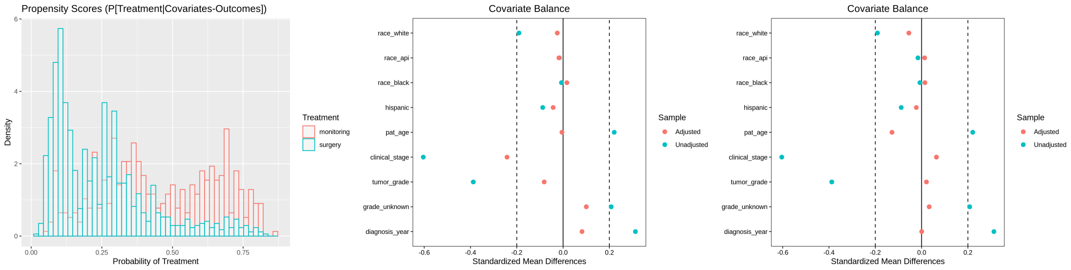

(b) Surgery vs Monitoring (prostate, glmnet)

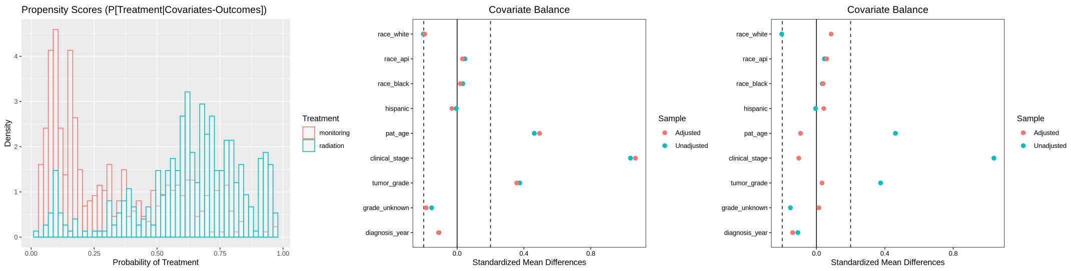

(c) Monitoring vs Radiation (prostate, glmnet)

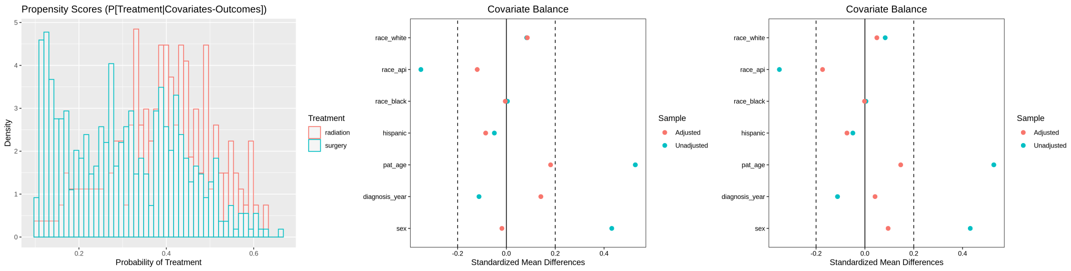

(d) Surgery vs Radiation (lung, grf)

Supplementary Figure 2: Supplementary plots to the `structured` results presented in Figure 3. (left) Propensity score plot with `structured`. (middle) Covariate balance plot for matching. (right) Covariate balance plots for IPTW.

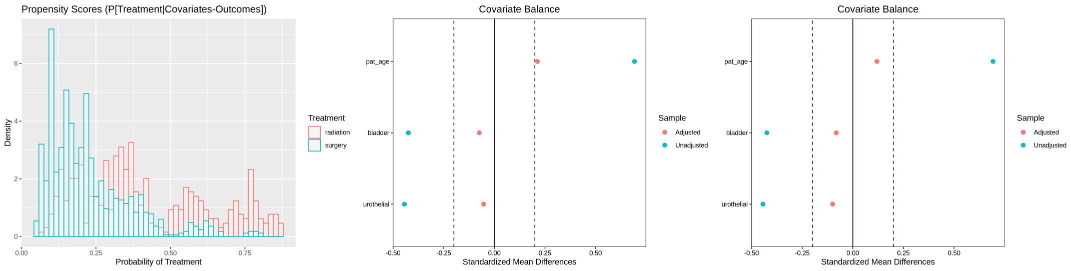

(a) Surgery vs Radiation (prostate, grf)

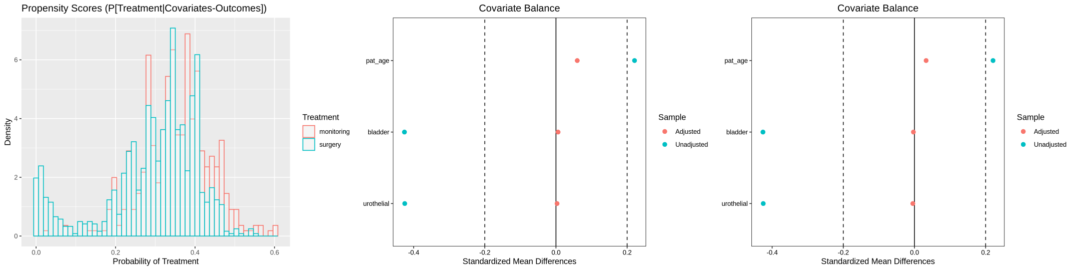

(b) Surgery vs Monitoring (prostate, glmnet)

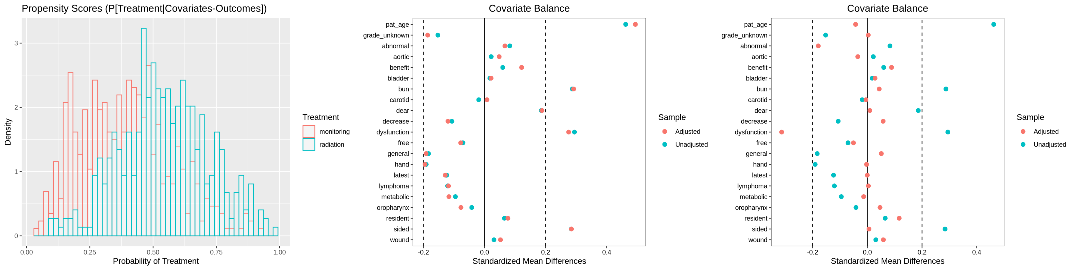

(c) Monitoring vs Radiation (prostate, glmnet)

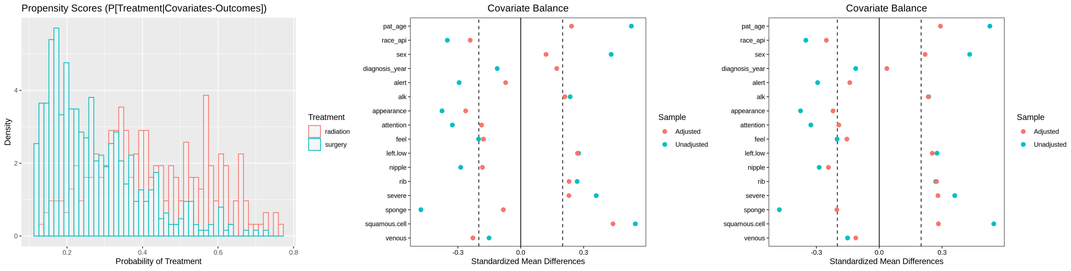

(d) Surgery vs Radiation (lung, grf)

Supplementary Figure 3: Supplementary plots to the intersect results presented in Figure 3. (left) Propensity score plot with intersect. (middle) Covariate balance plot for matching. (right) Covariate balance plots for IPTW.

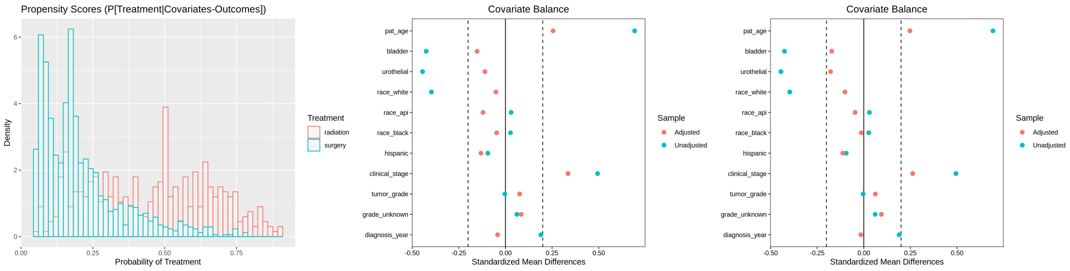

(a) Surgery vs Radiation (prostate, grf)

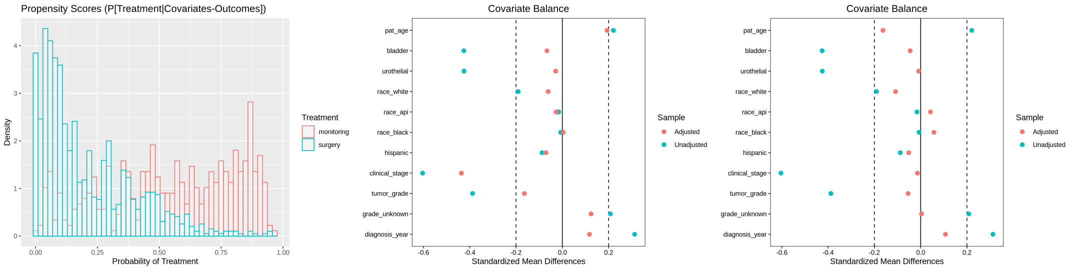

(b) Surgery vs Monitoring (prostate, glmnet)

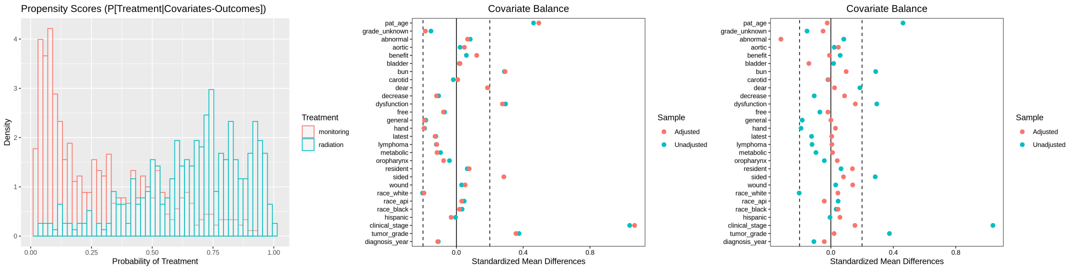

(c) Monitoring vs Radiation (prostate, glmnet)

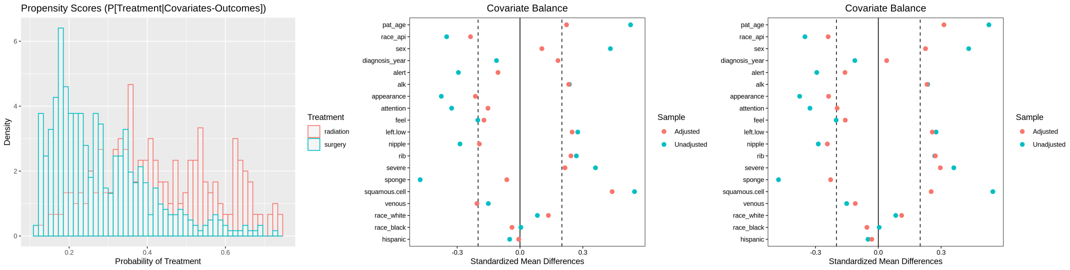

(d) Surgery vs Radiation (lung, grf)

Supplementary Figure 4: Supplementary plots to the struct+intersect results presented in Figure 3. (left) Propensity score plot with struct+intersect. (middle) Covariate balance plot for matching. (right) Covariate balance plots for IPTW.

radiation vs. monitoring for prostate cancer. In Supplementary Table 5, we show the R^2 values for surgery vs. radiation for NSCLC.

Supplementary Table 2: R^2 correlation of the struct+intersect covariates of surgery vs. radiation for prostate cancer.

	struct:patient_age	text:bladder	text:urothelial	struct:race_white	struct:race_api	struct:race_black	struct:hispanic	struct:clinical_stage	struct:tumor_grade	struct:grade_unknown	struct:diagnosis_year
struct:patient_age		0.04	0.01	0.00	0.00	0.00	0.01	0.00	0.00	0.00	0.02
text:bladder	0.04		0.47	0.01	0.00	0.00	0.00	0.04	0.03	0.00	0.00
text:urothelial	0.01	0.47		0.01	0.00	0.00	0.00	0.04	0.03	0.00	0.00
struct:race_white	0.00	0.01	0.01		0.22	0.07	0.05	0.00	0.00	0.00	0.03
struct:race_api	0.00	0.00	0.00	0.22		0.00	0.01	0.00	0.00	0.00	0.00
struct:race_black	0.00	0.00	0.00	0.07	0.00		0.00	0.00	0.00	0.00	0.00
struct:hispanic	0.01	0.00	0.00	0.05	0.01	0.00		0.00	0.00	0.00	0.00
struct:clinical_stage	0.00	0.04	0.04	0.00	0.00	0.00	0.00		0.04	0.01	0.03
struct:tumor_grade	0.00	0.03	0.03	0.00	0.00	0.00	0.00	0.04		0.51	0.21
struct:grade_unknown	0.00	0.00	0.00	0.00	0.00	0.00	0.00	0.01	0.51		0.02
struct:diagnosis_year	0.02	0.00	0.00	0.03	0.00	0.00	0.00	0.03	0.21	0.02	

Supplementary Table 3: R^2 correlation of the struct+intersect covariates of surgery vs. monitoring for prostate cancer.

	struct:patient_age	text:carotid	text:resident	struct:race_white	struct:race_api	struct:race_black	struct:hispanic	struct:clinical_stage	struct:tumor_grade	struct:grade_unknown	struct:diagnosis_year
struct:patient_age		0.02	0.00	0.00	0.01	0.01	0.00	0.04	0.01	0.01	0.07
text:carotid	0.02		0.00	0.00	0.00	0.00	0.00	0.00	0.01	0.00	0.00
text:resident	0.00	0.00		0.00	0.00	0.01	0.00	0.00	0.00	0.00	0.00
struct:race_white	0.00	0.00	0.00		0.17	0.06	0.02	0.00	0.00	0.00	0.00
struct:race_api	0.01	0.00	0.00	0.17		0.00	0.00	0.00	0.00	0.00	0.01
struct:race_black	0.01	0.00	0.01	0.06	0.00		0.00	0.00	0.00	0.00	0.00
struct:hispanic	0.00	0.00	0.00	0.02	0.00	0.00		0.00	0.00	0.00	0.00
struct:clinical_stage	0.04	0.00	0.00	0.00	0.00	0.00	0.00		0.05	0.01	0.06
struct:tumor_grade	0.01	0.01	0.00	0.00	0.00	0.00	0.00	0.05		0.66	0.22
struct:grade_unknown	0.01	0.00	0.00	0.00	0.00	0.00	0.00	0.01	0.66		0.04
struct:diagnosis_year	0.07	0.00	0.00	0.00	0.01	0.00	0.00	0.06	0.22	0.04	

Supplementary Table 4: R^2 correlation of the struct+intersect covariates of radiation vs. monitoring for prostate cancer.

	struct:patient_age	text:carotid	text:resident	struct:race_white	struct:race_api	struct:race_black	struct:hispanic	struct:clinical_stage	struct:tumor_grade	struct:grade_unknown	struct:diagnosis_year
struct:patient_age		0.02	0.00	0.00	0.01	0.01	0.00	0.04	0.01	0.01	0.07
text:carotid	0.02		0.00	0.00	0.00	0.00	0.00	0.00	0.01	0.00	0.00
text:resident	0.00	0.00		0.00	0.00	0.01	0.00	0.00	0.00	0.00	0.00
struct:race_white	0.00	0.00	0.00		0.17	0.06	0.02	0.00	0.00	0.00	0.00
struct:race_api	0.01	0.00	0.00	0.17		0.00	0.00	0.00	0.00	0.00	0.01
struct:race_black	0.01	0.00	0.01	0.06	0.00		0.00	0.00	0.00	0.00	0.00
struct:hispanic	0.00	0.00	0.00	0.02	0.00	0.00		0.00	0.00	0.00	0.00
struct:clinical_stage	0.04	0.00	0.00	0.00	0.00	0.00	0.00		0.05	0.01	0.06
struct:tumor_grade	0.01	0.01	0.00	0.00	0.00	0.00	0.00	0.05		0.66	0.22
struct:grade_unknown	0.01	0.00	0.00	0.00	0.00	0.00	0.00	0.01	0.66		0.04
struct:diagnosis_year	0.07	0.00	0.00	0.00	0.01	0.00	0.00	0.06	0.22	0.04	

Supplementary Table 5: R^2 correlation of the struct+intersect covariates of surgery vs. radiation for NSCLC. Due to the size of the table, we've omitted the "struct:" and "text:" prefixes for the covariates.

	patient_age	race_api	sex	diagnosis_year	alert	alk	allergy	appearance	attention	cyst	discomfort	eye	fever	inguinal	nipple	rib	severe	silhouette	sponge	squamous	race_white	race_black	hispanic
patient_age		0.00	0.01	0.04	0.01	0.00	0.00	0.02	0.01	0.00	0.01	0.00	0.00	0.00	0.00	0.00	0.01	0.00	0.01	0.00	0.00	0.00	0.00
race_api	0.00		0.00	0.03	0.02	0.00	0.00	0.03	0.00	0.00	0.00	0.00	0.00	0.01	0.00	0.01	0.00	0.00	0.00	0.01	0.30	0.00	0.01
sex	0.01	0.00		0.04	0.01	0.01	0.01	0.01	0.01	0.02	0.00	0.01	0.00	0.02	0.02	0.00	0.00	0.00	0.01	0.01	0.02	0.00	0.00
diagnosis_year	0.04	0.03	0.04		0.11	0.01	0.03	0.02	0.01	0.00	0.00	0.01	0.01	0.01	0.00	0.01	0.00	0.00	0.01	0.01	0.01	0.00	0.00
alert	0.01	0.02	0.01	0.11		0.00	0.02	0.04	0.01	0.00	0.01	0.01	0.05	0.00	0.00	0.00	0.00	0.00	0.02	0.00	0.00	0.00	0.00
alk	0.00	0.00	0.01	0.01	0.00		0.00	0.00	0.00	0.00	0.00	0.00	0.00	0.00	0.00	0.00	0.01	0.00	0.00	0.00	0.00	0.00	0.00
allergy	0.00	0.00	0.01	0.03	0.02	0.00		0.02	0.00	0.00	0.00	0.01	0.04	0.00	0.02	0.00	0.00	0.00	0.00	0.00	0.00	0.00	0.00
appearance	0.02	0.03	0.01	0.02	0.04	0.00	0.02		0.00	0.00	0.00	0.00	0.03	0.00	0.00	0.00	0.00	0.00	0.00	0.00	0.00	0.00	0.00
attention	0.01	0.00	0.01	0.01	0.01	0.00	0.00	0.00		0.00	0.00	0.00	0.00	0.00	0.00	0.00	0.00	0.00	0.00	0.00	0.00	0.00	0.00
cyst	0.00	0.00	0.02	0.00	0.00	0.00	0.00	0.00	0.00		0.00	0.00	0.00	0.00	0.01	0.00	0.00	0.00	0.00	0.00	0.01	0.00	0.00
discomfort	0.01	0.00	0.00	0.00	0.01	0.00	0.00	0.00	0.00	0.00		0.00	0.00	0.00	0.00	0.01	0.01	0.00	0.00	0.00	0.00	0.00	0.00
eye	0.00	0.00	0.01	0.01	0.01	0.00	0.01	0.00	0.00	0.00	0.00		0.00	0.00	0.00	0.00	0.00	0.00	0.00	0.00	0.00	0.00	0.00
fever	0.00	0.00	0.00	0.01	0.05	0.00	0.04	0.03	0.00	0.00	0.00	0.00		0.01	0.00	0.00	0.00	0.00	0.00	0.00	0.00	0.00	0.00
inguinal	0.00	0.01	0.02	0.01	0.00	0.00	0.00	0.00	0.00	0.00	0.00	0.00	0.01		0.00	0.00	0.00	0.00	0.00	0.01	0.00	0.00	0.00
nipple	0.00	0.00	0.02	0.00	0.00	0.00	0.02	0.00	0.00	0.01	0.00	0.00	0.00	0.00		0.00	0.00	0.00	0.00	0.00	0.01	0.00	0.00
rib	0.00	0.01	0.00	0.01	0.00	0.00	0.00	0.00	0.00	0.00	0.01	0.00	0.00	0.00	0.00		0.00	0.00	0.00	0.00	0.00	0.00	0.00
severe	0.01	0.00	0.00	0.00	0.00	0.01	0.00	0.00	0.00	0.00	0.01	0.00	0.00	0.00	0.00	0.00		0.00	0.01	0.00	0.00	0.00	0.01
silhouette	0.00	0.00	0.00	0.00	0.00	0.00	0.00	0.00	0.00	0.00	0.00	0.00	0.00	0.00	0.00	0.00	0.00		0.00	0.00	0.00	0.00	0.00
sponge	0.01	0.00	0.01	0.01	0.02	0.00	0.00	0.00	0.00	0.00	0.00	0.00	0.00	0.00	0.00	0.00	0.01	0.00		0.00	0.01	0.00	0.00
squamous	0.00	0.01	0.01	0.01	0.00	0.00	0.00	0.00	0.00	0.00	0.00	0.00	0.00	0.01	0.00	0.00	0.00	0.00	0.00		0.00	0.00	0.00
race_white	0.00	0.30	0.02	0.01	0.00	0.00	0.00	0.00	0.00	0.01	0.00	0.00	0.00	0.00	0.01	0.00	0.00	0.00	0.01	0.00		0.04	0.04
race_black	0.00	0.00	0.00	0.00	0.00	0.00	0.00	0.00	0.00	0.00	0.00	0.00	0.00	0.00	0.00	0.00	0.00	0.00	0.00	0.00	0.04		0.00
hispanic	0.00	0.01	0.00	0.00	0.00	0.00	0.00	0.00	0.00	0.00	0.00	0.00	0.00	0.00	0.00	0.00	0.01	0.00	0.00	0.00	0.04	0.00	
