# Supplementary material for: Uncovering interpretable potential confounders in electronic medical records
Source: Nat Commun. 2022 Feb 23;13:1014. doi: 10.1038/s41467-022-28546-8 (PMC8866497; doi:10.1038/s41467-022-28546-8)
Supplement: Supplementary file 2 — Reporting Summary [file 41467_2022_28546_MOESM2_ESM.pdf]

nature portfolio

Corresponding author(s): Jiaming Zeng
Last updated by author(s): Sep 11, 2021

# Reporting Summary

Nature Portfolio wishes to improve the reproducibility of the work that we publish. This form provides structure for consistency and transparency in reporting. For further information on Nature Portfolio policies, see our Editorial Policies and the Editorial Policy Checklist.

## Statistics

For all statistical analyses, confirm that the following items are present in the figure legend, table legend, main text, or Methods section.

| n/a | Confirmed | |
|---|---|---|
| ☐ | ☒ | The exact sample size ($n$) for each experimental group/condition, given as a discrete number and unit of measurement |
| ☐ | ☒ | A statement on whether measurements were taken from distinct samples or whether the same sample was measured repeatedly |
| ☐ | ☒ | The statistical test(s) used AND whether they are one- or two-sided<br>*Only common tests should be described solely by name; describe more complex techniques in the Methods section.* |
| ☐ | ☒ | A description of all covariates tested |
| ☐ | ☒ | A description of any assumptions or corrections, such as tests of normality and adjustment for multiple comparisons |
| ☐ | ☒ | A full description of the statistical parameters including central tendency (e.g. means) or other basic estimates (e.g. regression coefficient) AND variation (e.g. standard deviation) or associated estimates of uncertainty (e.g. confidence intervals) |
| ☐ | ☒ | For null hypothesis testing, the test statistic (e.g. $F$, $t$, $r$) with confidence intervals, effect sizes, degrees of freedom and $P$ value noted<br>*Give P values as exact values whenever suitable.* |
| ☒ | ☐ | For Bayesian analysis, information on the choice of priors and Markov chain Monte Carlo settings |
| ☒ | ☐ | For hierarchical and complex designs, identification of the appropriate level for tests and full reporting of outcomes |
| ☒ | ☐ | Estimates of effect sizes (e.g. Cohen's $d$, Pearson's $r$), indicating how they were calculated |

*Our web collection on statistics for biologists contains articles on many of the points above.*

## Software and code

Policy information about availability of computer code

| | |
|---|---|
| Data collection | We pull the data from the Stanford Cancer Institute through Google Cloud GCP. |
| Data analysis | We used Python 3 and R 4.0 to analyze the code. For python, we used pandas v1.4.0, NLTK v2.0 , scispaCy v.0.4.0 , and scikit-learn v0.24.0 to process the data. The processed data is then analyzed using R with the software packages survival v3.2, glmnet v4.1, grf v2.0.2, MatchIt v4.3.2. The complete pipeline for R analysis is available at https://github.com/jmzeng/interpretable-potential-confounders. |

For manuscripts utilizing custom algorithms or software that are central to the research but not yet described in published literature, software must be made available to editors and reviewers. We strongly encourage code deposition in a community repository (e.g. GitHub). See the Nature Portfolio guidelines for submitting code & software for further information.

## Data

Policy information about availability of data

All manuscripts must include a data availability statement. This statement should provide the following information, where applicable:

- Accession codes, unique identifiers, or web links for publicly available datasets
- A description of any restrictions on data availability
- For clinical datasets or third party data, please ensure that the statement adheres to our policy

The datasets analyzed for the study are not publicly available. We extracted the data from the Stanford Cancer Institute Research Database, the California Cancer Registry, and the Epic System. The EHR data cannot be redistributed to researchers other than those approved through the Stanford Institutional Review Board and those who have obtained a Material Transfer Agreement. We have therefore given detailed description of our data selection and processing pipeline in the Methods section. To request access to the data, please contact Jiaming Zeng at jiaming@alumni.stanford.edu.

# Field-specific reporting

Please select the one below that is the best fit for your research. If you are not sure, read the appropriate sections before making your selection.

☐ Life sciences ☒ Behavioural & social sciences ☐ Ecological, evolutionary & environmental sciences

For a reference copy of the document with all sections, see nature.com/documents/nr-reporting-summary-flat.pdf

# Behavioural & social sciences study design

All studies must disclose on these points even when the disclosure is negative.

| | |
|---|---|
| Study description | The study is uses observational electronic medical records from the Stanford Cancer Institute and the California Cancer Registry. The study includes both qualitative, quantitative, and textual data. |
| Research sample | Our data is sourced from the Stanford Cancer Institute Research Database, the California Cancer Registry, and the Epic System. We sample local prostate and lung cancer patients from the Stanford Hospital who have received the treatments of interest. For prostate cancer, we focused on active monitoring, surgery, and radiation. For lung cancer, we sampled for stage I non-small cell lung cancer patients who have receiver surgery or radiation. We chose this study population for comparison to existing randomized control trials on localized prostate and lung cancer. |
| Sampling strategy | In addition to the sample we selected above, we sampled patients based on the quality and quantity of data available. Since our goal is to uncover potential confounders from the clinical text, we filtered for patients who have at least one clinical note available 1-2 months before the administered treatment. |
| Data collection | The Stanford Cancer Institute data have been recorded and curated by Stanford Hospital staff through the computer. The California Cancer Registry is manually curated by the registry staff through computer. The data from the Epic system was also recorded through the computer. The researcher was blind to the experimental conditions and/or study hypothesis. |
| Timing | The localized prostate cancer data includes patient diagnosed from 2008-2017. The localized lung cancer data includes patient diagnosed from 2000-2017. |
| Data exclusions | We excluded patients who do not have our treatment of interest or do not have enough clinical notes for valid analysis. We also select for patients who have selected at least 6 months past diagnosis to avoid immortal time bias. |
| Non-participation | No active participants were included in the study. We focus on retrospective data. |
| Randomization | We use observational data with no randomization. We control for confounding using statistical methods with structured and unstructured covariates from the medical records. |

# Reporting for specific materials, systems and methods

We require information from authors about some types of materials, experimental systems and methods used in many studies. Here, indicate whether each material, system or method listed is relevant to your study. If you are not sure if a list item applies to your research, read the appropriate section before selecting a response.

## Materials & experimental systems

| n/a | Involved in the study |
|---|---|
| ☒ | ☐ Antibodies |
| ☒ | ☐ Eukaryotic cell lines |
| ☒ | ☐ Palaeontology and archaeology |
| ☒ | ☐ Animals and other organisms |
| ☒ | ☐ Human research participants |
| ☒ | ☐ Clinical data |
| ☒ | ☐ Dual use research of concern |

## Methods

| n/a | Involved in the study |
|---|---|
| ☒ | ☐ ChIP-seq |
| ☒ | ☐ Flow cytometry |
| ☒ | ☐ MRI-based neuroimaging |
